# Tetrodotoxins Occurrence in Non-Traditional Vectors of the North Atlantic Waters (Portuguese Maritime Territory, and Morocco Coast)

**DOI:** 10.3390/toxins11060306

**Published:** 2019-05-29

**Authors:** Marisa Silva, Inés Rodríguez, Aldo Barreiro, Manfred Kaufmann, Ana Isabel Neto, Meryem Hassouani, Brahim Sabour, Amparo Alfonso, Luis M. Botana, Vitor Vasconcelos

**Affiliations:** 1 Department of Biology, Faculty of Sciences, University of Porto, Rua do Campo Alegre, 4619-007 Porto, Portugal; aldo.barreiro@gmail.com (A.B.); vmvascon@fc.up.pt (V.V.); 2 Interdisciplinary Center of Marine and Environmental Research-CIMAR/CIIMAR, University of Porto, Novo Edificio do Terminal de Cruzeiros do Porto de Leixões, Avenida General Norton de Matos, 4450-208 S/N Matosinhos, Portugal; mkaufmann@ciimar.up.pt (M.K.); ana.im.neto@uac.pt (A.I.N.); 3 Laboratorio CIFGA S.A., Plaza de Santo Domingo no. 20, 5a planta, 27001 Lugo, Spain; inesrguez@cifga.es; 4 Department of Pharmacology, Faculty of Veterinary, University of Santiago of Compostela, 27002 Lugo, Spain; amparo.alfonso@usc.es (A.A.); Luis.Botana@usc.es (L.M.B.); 5 Marine Biology Station of Funchal, Faculty of Life Sciences, University of Madeira, 9000-107 Funchal, Portugal; 6 Center of Interdisciplinary Marine and Environmental Research of Madeira—CIIMAR-Madeira, Edif. Madeira Tecnopolo, Caminho da Penteada, 9020-105 Funchal, Portugal; 7 cE3c/GBA—Centre for Ecology, Evolution and Environmental Changes/Azorean Biodiversity Group and Department of Biology, Faculty of Sciences and Technology, University of Azores, 9501-801 Ponta Delgada, São Miguel, Azores, Portugal; 8 Phycology Research Unit—Biotechnology, Ecosystems Ecology and Valorization Laboratory, Faculty of Sciences El Jadida, University Chouaib Doukkali, BP20 El Jadida, Morocco; hassouani@hotmail.com (M.H.); sabour.b@ucd.ac.ma (B.S.)

**Keywords:** tetrodotoxin, new vectors, North Atlantic Waters

## Abstract

Tetrodotoxin (TTX) is a potent alkaloid typically from tropical ecosystems, but in the last decade its presence has been more pronounced in the temperate waters of the Atlantic. In its last scientific opinion, the European Food Safety Authority (EFSA) stressed the need for data regarding TTX prevalence in European waters. To address EFSA’s concerns, benthic organisms such as mollusks, crustaceans, echinoderms and fish with different feeding habits were collected along the Portuguese continental coast, islands (São Miguel, Azores, and Madeira) and the northwestern Moroccan coast. A total of 165 samples were analyzed by ultra high performance liquid chromatography high resolution mass spectrometry (UHPLC-HRMS) and ultra high performance chromatography mass spectrometry (UHPLC-MS/MS). Geographical tendencies were detected as follows, by descending order: S. Miguel Island (Azores), Moroccan coast, Madeira Island and Portuguese continental coast. The toxin amounts detected were significant, above the Dutch limit value established in 2017, showing the importance and the need for continuity of these studies to gain more knowledge about the prevalence of these toxins, unraveling new vectors, in order to better assess human health risk. This work represents a general overview of new TTX bearers (7) most of them in gastropods (*Patella depressa*, *Nucella lapillus*, *Onchidella celtica* and *Aplysia depilans*), followed by echinoderms (*Echinus esculentus* and *Ophidiaster ophidianus*) and puffer fish *Sphoeroides marmoratus*.

## 1. Introduction

Tetrodotoxin (TTX) is an extremely potent alkaloid typical from warm ecosystems that over recent years became more frequent in temperate waters [1,2,3]. Named after the Tetraodontidae puffer fish family where it was first isolated, TTX was later identified also in different taxa not close related (from bacteria; marine invertebrates; terrestrial and marine vertebrates) [4,5]. The ubiquity of TTX is due to its exogenous origin, bacteria from the different phyla (Proteobacteria, Firmicutes, Bacterioides and Actinobacteria) associated with dinoflagellate blooms of *Prorocentrum* have been pointed to as potential producers [6,7,8]. 

Chemically, TTX is a crystalline weakly basic heterocyclic molecule with six hydroxyl groups, a pyridine ring with additional fused ring systems and a guanidinium group, positively charged at a physiological pH, with a molecular formula of C_11_H_17_O_8_N_3_ (Table 1) [1]. This powerful alkaloid exhibits its action by binding selectively and extracellularly to receptor-site 1 of voltage-gated sodium channels (Na_v_s), once it mimics the sodium hydrated cation, occluding the outer pore. Thus, avoiding the access of monovalent cations to the pore, the generation and propagation of the action potential is blocked, inhibiting the communication between electrically excitable cells in muscle and nerve tissues, causing paralysis which results in death by cardio-respiratory failure [9,10,11]. About 30 analogues have been described to date, whose toxicity varies with structure implying their greater or lesser affinity for their molecular target [12]. The rise of water temperature together with anthropological intervention aided the migration and establishment of this neurotoxin typically from tropical ecosystems, the Pacific and Indian oceans, into European waters, where it has become more prevalent in the last decade [13,14,15,16,17]. Regarding consumer protection in the EU, regulations 853/2004/EC and 854/2004/EC were published in 2004, preventing fish species reported as TTX-bearers (Tetraodontidae, Canthigasteridae, Molidae and Diodontidae) from market placement [18,19]; the Netherlands being the pioneer in including the scrutinizing of this toxin in its monitoring program, establishing 44 µg TTX equivalents/kg of shellfish as a safe concentration [20]. Still, more data are needed in order to better protect consumer health. In EFSA’s latest opinion it’s stated that more occurrence data on TTX and its derivatives in edible parts of gastropods and bivalves are necessary in order to complement a reliable exposure assessment, since it was not possible to include gastropods in the risk characterization due to insufficient studies [21]. The primary aim of this work was to screen the presence of TTX in different benthic marine species along the Portuguese continental and archipelagos and western Moroccan coasts, thus unraveling its prevalence and geographical trends. We hope to bridge and to contribute to EFSA’s recommendations, towards the development of effective and robust monitoring programs.

## 2. Results and Discussion

**Figure 1:**
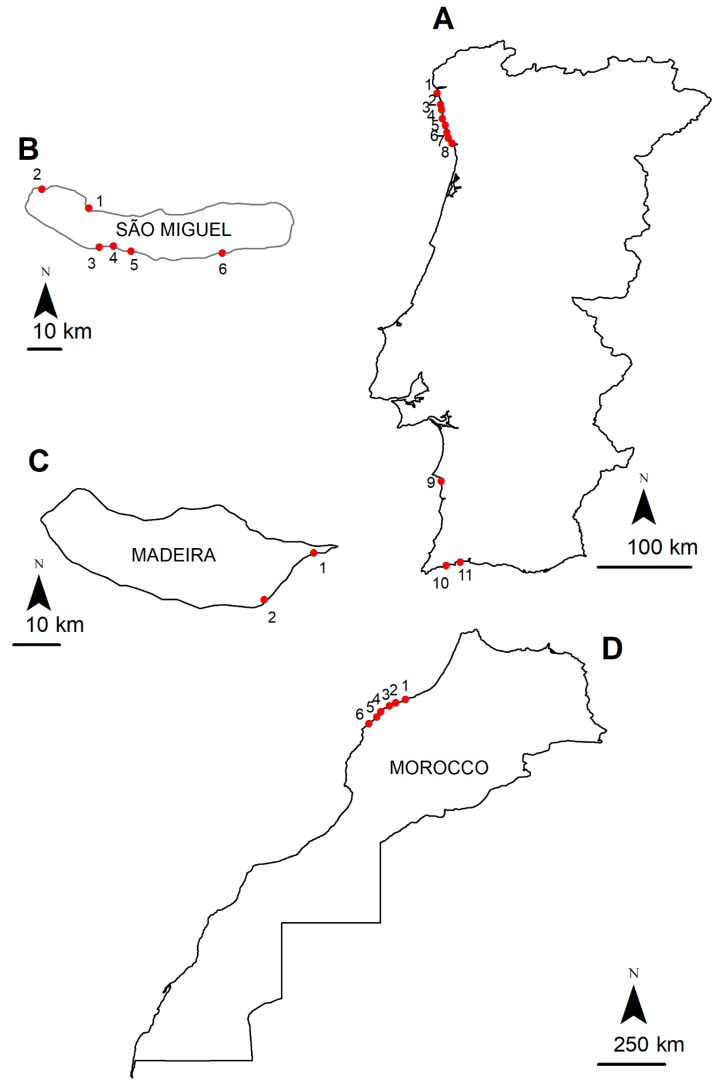
Location of the sampling points: (**A**) Portuguese continental coast: 1, Esposende; 2, Póvoa do Varzim; 3, Angeiras; 4, Memória; 5, Pedra de Jacó; 6, Pêlo Negro; 7, Valadares; 8, Ovar; 9, Porto Côvo; 10, Camilo; 11, Luz. (**B**) Madeira Island coast: 1, Caniçal and 2, Reis Magos. (**C**) São Miguel Island coast, Azores archipelago: 1, Cruzeiro; 2, Mosteiros; 3, Étar de Ponta Delgada; 4, São Roque; 5, Lagoa; 6, Caloura. (**D**) Northwestern Moroccan coast: 1, Casablanca Corniche; 2, El Jadida Haras; 3, El Jadida Sâada; 4, Sidi Bouzid; 5, Mrizika; 6, Oualidia.

A total of 165 samples collected from 25 different sampling points (Figure 1) were screened using the techniques of ultra high performance chromatography mass spectrometry (UHPLC-MS/MS) and ultra high performance liquid chromatography high resolution mass spectrometry (UHPLC-HRMS) [8,22]. These two techniques are complementary when performing a complete and undoubted identification of TTX analogues when standards are not available. UHPLC-MS/MS in product ion scan (PIS) mode allowed to check the mass fragmentation pattern of this family of compounds and HRMS was used to obtain the exact mass of each compound. Finally, the positive samples were quantified by UHPLC-MS/MS in multiple reaction monitoring (MRM) mode using a TTX standard. About 30 species of edible, with commercial interest, and non-edible benthic organisms were collected in the intertidal zone and by Self-Contained Underwater Breathing Apparatus (SCUBA) diving, with the aim of searching new vectors and determining the prevalence of TTXs in the food web: bivalves (mussels), gastropods (limpets, sea-snails, sea-slugs), echinoderms (starfish, sea-urchins, sea-cucumbers), crustaceans (barnacles) and fish (puffer). Sampling points and collected species are described in detail in the Materials and Methods section.

**Figure 2:**
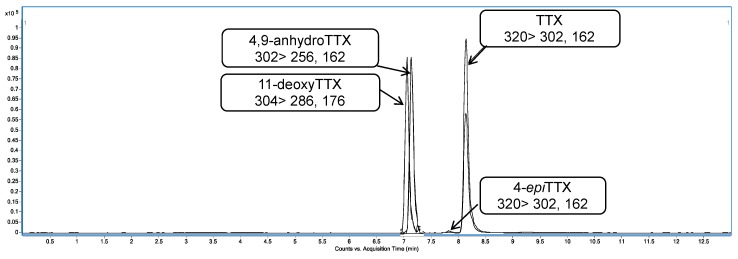
Multiple reaction monitoring (MRM) chromatogram obtained in positive mode of tetrodotoxin (TTX) standard.

**Figure 3:**
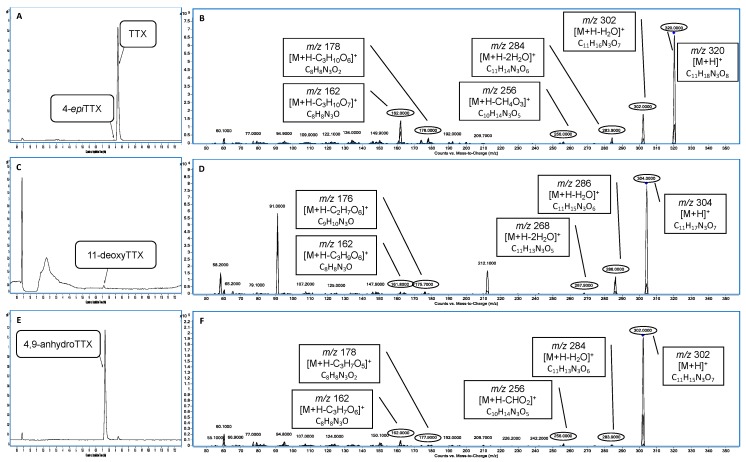
Chromatogram and mass spectrum of TTX standard. (**A**) Chromatogram of TTX and 4-*epi*TTX obtained by PIS. (**B**) Mass fragmentation pattern of TTX and 4-*epi*TTX. (**C**) Chromatogram of 11-deoxyTTX obtained by PIS. (**D**) Mass fragmentation pattern of 11-deoxyTTX. (**E**) Chromatogram of 4,9-anhydroTTX obtained by PIS. (**F**) Mass fragmentation pattern of 4,9-anhydroTTX.

In the present study, a variety of samples from different animals were analyzed using the UHPLC-MS/MS method after the extraction procedure. Initially, samples were analyzed in MRM mode for 18 TTX analogues described in the literature [1] and some compounds of this family were detected in several samples. This preliminary analysis showed many positive samples for TTX plus analogues. One of the great challenges of the emerging toxins is the lack of reference material, as far as TTX is concerned, therefore only the analogues present in the standard (TTX, 4,9-anhydroTTX, 4-*epi*TTX and 11-deoxyTTX) could be confirmed (Figure 2). However, all positive extracts presented several TTX analogues. To confirm these positive results, their mass fragmentation pattern was studied [8,23,24]. The fragmentation pathway of TTX standard shows two losses of water and *m*/*z* 256, *m*/*z* 178 and *m*/*z* 162 as product ions (Figure 3) [8,24,25].

In this way, TTXs analogues were confirmed in several samples. As shown in Table 2, a loss of water and *m*/*z* 178 (or 176) and *m*/*z* 162 are the product ions of TTX analogues detected in positive samples.

At the same time, the suspicious positive samples were also analyzed using High Resolution Mass Spectrometry (HRMS) to confirm and identify TTX analogues. The mass fragmentation pathway of TTX analogues has been reported in many LC-HRMS studies [8,26]. In the current study the exact mass of TTX analogues was determined by HRMS using the formula predictor software to characterize and predict the molecular formula of these unknown compounds. In this way, elemental composition, theoretical value *m*/*z*, experimental *m*/*z* and mass errors in mDa for TTX analogues were obtained after MS^1^ spectra (Table 3, Table 4 and Table 5).

Once the presence of TTX analogues was verified, the toxin amount was quantified using LC-MS/MS in MRM mode (Table 3, Table 4 and Table 5). In order to calculate the concentrations of TTXs, the calibration curve was done with TTX standard, assuming that related analogues would give a similar response of TTX. The quantification was done using MRM acquisition in positive mode. A seven-point calibration curve with range between 1.5 ng/mL–100 ng/mL was used (R^2^ = 0.999). The limit of detection (LOD) was 1.64 µg/Kg and the limit of quantification (LOQ) 5.46 µg/Kg.

### 2.1. Portuguese Continental Coast

In a total of 62 analyzed samples collected between September 2011 and January 2012 at the 11 sampling points (Figure 1), 8 positive results were detected, all below the LOQ (Table 3).

Regarding positive hits for TTXs, despite not quantifiable, we count three first reports of this group of toxins, two in gastropods, *P. depressa* and *N. lapillus,* for the analogs 11-oxoTTX and trideoxyTTX, respectively and one in echinoderms, *E. esculentus* positive for 11-oxoTTX. In our former work, the analogue trideoxyTTX was identified only in one sample of the species *C. lampas* [16]; in the present study, this analogue was the most common along the Portuguese coast, being present in five of seven positive organisms, all gastropods: *P. lineatus*, *C. lampas*, *N. lapillus*, *G. umbilicalis*.

The analogue *m*/*z* 336 was detected in *P. depressa* (225), *E. esculentus* (229_2) and *P. lineatus* (239). The fragmentation pattern of this molecule shows *m*/*z* 318, corresponding to [M+H−H_2_O]^+^, *m*/*z* 176 and *m*/*z* 162 [31]. After HRMS (Table 3) the analogue 11-oxoTTX can be proposed as *m*/*z* 336 with a small error (−3.8 and −1.8 mDa).

*P. lineatus* (226), *C. lampas* (232_2 and 232_3), *N. lapillus* (236) and *G. umbilicalis* (240) presented a TTX analogue with *m*/*z* 272 and a fragmentation pathway with loss of water, *m*/*z* 254, and the product ions *m*/*z* 178 and *m*/*z* 162 [24]. The TTX analogue with an elemental composition of C_11_H_17_N_3_O_5_ is suggested (error of 0.9–1.1 mDa) after HRMS analysis (Table 3).

Finally, the samples were quantified by MRM using TTX standard, assuming that the TTX analogues have a similar response. In this case, the quantification was not possible since the amount present in the samples was below the LOQ.

### 2.2. Madeira Island (Madeira Archipelago) 

From a total of 23 samples harvested in two sampling points (Figure 1) during the summer of 2012, we reported zero positive hits for TTX and its derivatives.

### 2.3. São Miguel Island (Azores Archipelago)

São Miguel island, Azores, was screened during June 2013. From the 39 harvested samples along 6 sampling points (Figure 1), we detected 4 positive hits (Table 4).

Regarding toxin uptake, we found in São Miguel, Azores, a distinct toxin profile concerning positive taxa and detected toxins, also all detected values were above the current limit established in the Netherlands [20]. Starting with echinoderms, all positive samples were in the red velvet starfish, *O. ophidianus*, for an unknown TTX analogue since the molecule that follows TTX mass fragmentation pattern, further studies are needed. The sample of *S. marmoratus* was analyzed per tissue (muscle, gonads and liver), since the specimen belongs to the Tetraodontidae family, and there was sufficient extractable biomass. Discussing uptake by tissue, the values detected curiously increased in the following order: liver, muscle, gonads. Surprisingly, the liver had half the values detected in the muscle and ten times less than in the gonads, which deviates from the typical accumulation pattern for this family of fish [34,35]. However, a study performed in the United States of America, between 2002 and 2004, showed the same uptake pattern, for the saxitoxin group, in the species *Sphoeroides nephelus*, detecting concentrations in the muscle 5 to 20 fold higher in comparison to the liver [36]. Concerning toxic profile, all tissues were positive for TTX, 4-*epi*TTX, monodeoxyTTX, 4,9-anhydroTTX, dideoxyTTX, trideoxyTTX and anhydrotrideoxyTTX, being the later derivative not detected in the liver tissue.

In comparison with other works regarding TTX concentrations in starfish [37,38], though they are a distinct ecotype and species, the present measured concentrations are higher. Though chronologically distant from the Japanese studies, in the present work, the considerable detected amounts could be explained by the rise of water temperature. In a simplistic approach, it has been shown that seawater temperature warming correlates with the global warming phenomenon; it was also proved that TTX production has a positive relationship with water temperature [39]. Other studies concerning other emergent toxin, ciguatera, predict that global warming is expected to be the catalyst for the increment of the incidence and prevalence of poisoning cases [40,41]. From our first report in 2012 on this neurotoxin group in Portugal, Angeiras was the northernmost point of TTX in Europe [16]. The latest reports corroborate this trend, since the last work points to its presence in England [42]. It is noteworthy, that this is the first report of TTXs in both species: *O. ophidianus* and *S. marmoratus*.

Three samples of *O. ophidianus* presented two TTX analogues with *m*/*z* 290 and different retention times (3.1 min and 4.6 min). The fragmentation mass pattern of all of them showed a loss of water and *m*/*z* 176 and *m*/*z* 162 product ions, as TTX. When these samples were analyzed using HRMS, a high error was observed compared to the 11-norTTX theoretical mass. Therefore, it can be concluded that these two TTX analogues have other elemental compositions different from 11-norTTX. Being aware of the error, and since it is in the same species, it could be due to matrix composition and the feeding habits of this particular species [32]. Further studies are needed.

The female fish, *S. marmoratus,* was divided in three different samples, gonads (438 G), liver (438 F), and muscle (438 M). Twelve analogues were detected in these samples, *m*/*z* 320 (TTX and 4-*epi*TTX), *m*/*z* 304 (11-deoxyTTX and other three deoxyTTX analogues), *m*/*z* 302 (4,9-anhydroTTX), *m*/*z* 288, *m*/*z* 272 (three analogues) and *m*/*z* 254. The fragmentation pathway of these peaks is similar to TTX, with a loss of water, *m*/*z* 178 (or 176) and *m*/*z* 162 product ions. Next, these samples were studied using HRMS (Table 4). As it was expected, TTX and 4-*epi*TTX (*m*/*z* 320 ions), 4,9-anhydroTTX (*m*/*z* 302) were confirmed with small errors (<2.5 mDa). Two *m*/*z* 304 analogues with retention times of 6.5 and 7.7 min were identified as deoxyTTX (error of 1-3.9 mDa). Another analogue, *m*/*z* 288 was recognized as dideoxyTTX with a retention time 6.4 min and an error of −3.3 mDa. Compound *m*/*z* 272 was detected in three peaks, retention times of 4.6, 4.8 min and 5 min. These three molecules could be associated with an elemental composition of C_11_H_17_N_3_O_5_, and errors 1.4, 1.3 and 4.3 mDa, respectively. Finally, TTX analogue *m*/*z* 254 was identified in *S. marmoratus* samples with an error of 0.6–1.3 mDa and elemental composition of C_11_H_15_N_3_O_4_.

### 2.4. Moroccan Coast

Thirty-eight samples were harvested along the northwestern Moroccan Coast for TTXs, during July 2013 at the 6 sampling points (Figure 1), with two positive hits for TTXs (Table 5).

Though the values detected were all below the reference value implemented in the Netherlands [20], all positives were in gastropods and first report of TTX in these two species, *A. depilans* and *O. celtica*, as TTX vectors. *O. celtica* had quantifiable amounts of TTX, 4-*epi*TTX and other TTX epimer, and *A. depilans* was positive below the limit of quantification for TTX and 4-*epi*TTX.

TTX and 4-*epi*TTX (*m*/*z* 320) were identified in *O. celtica* (474) and *A. depilans* (476) samples (Table 5). Besides, in sample 474 another molecule with *m*/*z* 320 and different retention time was detected. In this case, the fragmentation pattern was studied and the loss of water and *m*/*z* 178 and *m*/*z* 162 as product ions were obtained. This TTX analogue was analyzed by HRMS but due to the small quantity it could not be confirmed. As shown in Table 5, TTX and 4-*epi*TTX were confirmed by HRMS (error −0.6 mDa and 1.7 mDa). The low quantity of 476 sample prevents the confirmation of these compounds by HRMS.

### 2.5. Statistical Analysis

The TTX content per sample is a continuous variable with a large number of samples showing a value of 0 and potentially, overdispersion. Without overdispersion, the best models would be a zero-inflated or not zero-inflated model with Poisson distribution of model error. With overdispersion, we would need to change the error distribution of the model to quasipoisson or negative binomial. Then, in order to decide which statistical model to use, we first tested for overdispersion of our data.

Overdispersion was tested by fitting a generalized function overdispersion test, from the AER R package. This function was applied to a model with Poisson error distribution. In this model, the TTX content was the dependent variable, and the sampling site a factor with 4 levels (São Miguel island, Madeira, continental Portugal and Morocco). TTX content was rounded to appear as a discrete variable (needed for a model with Poisson error distribution). The overdispersion test results were non-significant (z = 1.22, *p* = 0.11), so it was possible to keep the Poisson model instead of using another one accounting for overdispersion. The goodness of fit of the model error to a Poisson distribution was also tested, with non-significant results (*p* = 1). Then, it was also not necessary to account for zero-inflation in our data.

An analysis of deviance applied to the previous model showed that ‘sampling site’ had a significant effect (χ^2^ = 882507, *p* < 0.001). The model coefficients are shown in Table 6, indicating that samples from São Miguel Island had the highest content of TTX, followed by Morocco, whereas Madeira and continental Portugal had the same coefficient value. Due to the large error of the estimate, these latter two coefficients were not significantly different from 0.

## 3. Conclusions

In summary, the primary aim of this work was to search for new vectors for TTXs off the Portuguese coast, islands and the northwestern coast of Morocco using UHPLC-HRMS and UHPLC-MS/MS techniques. We report for the first time seven new TTX bearers, 57% of them in gastropods (*P. depressa*, *N. lapillus*, *O. celtica* and *A. depilans*), 29% in echinoderms (*E. esculentus* and *O. ophidianus*) and 14% in fish (*S. marmoratus*). All the measurable concentrations ranged from 5 to 352,886 TTX eq µg/Kg of SM. Also, an unknown TTX analogue was detected only in the velvet starfish *O. ophidianus*, feeding habits can be responsible, but further studies are needed for confirmation.

Regarding geographical tendencies, S. Miguel Island in the Azores was the location with greatest propensity to find these biotoxin groups, followed by the Moroccan Coast; whereas Madeira and continental Portugal had the same coefficient value (≈0). Despite Madeira Island and Morocco having the same latitude, and the sampling being performed one year apart, the tendency values are quite different, which could be due to less anthropogenical interference in Madeira.

We hope this work represents a step forward in the emergent toxin phenomenon and in particularly in the tetrodotoxin theme in line with EFSA’s recommendations, stressing the importance of these works for better understanding this emergent subject and protection of public health.

## 4. Materials and Methods 

### 4.1. Sampled Points and Selected Species

TTX plus analogs were screened in 30 different species of benthic organisms, harvested from intertidal areas during low tide and in SCUBA diving expeditions. The areas surveyed comprised: the Portuguese continental coast (11 sampling points), Madeira and São Miguel Islands (8 sampling points) and the northwestern Moroccan coast (6 sampling points) (Table 7). The Portuguese continental coast was screened between September 2011 and January 2012, Madeira Island in September 2012, S. Miguel Island, Azores, and the northwestern coast of Morocco in June and July 2013 respectively. Different species of edible and non-edible organisms were screened to search for new potential vectors for TTXs: bivalves (*Mytilus galloprovincialis*, *Mytilus* spp.), sea-urchins (*Echinus esculentus*, *Arbacia lixula*, *Paracentrotus lividus*, *Sphaerechinus granularis*, *Diadema africanum*), sea-cucumber (*Holothuria (Platyperona) sanctori*), starfish (*Asterias rubens*, *Astropecten aranciacus*, *Marthasterias glacialis*, *Echinaster sepositus*, *Ophidiaster ophidianus*), crustaceans (*Pollicipes pollicipes*), gastropods (*Phorcus lineatus*, *Gibbula umbilicalis*, *Stramonita haemastoma*, *Charonia lampas*, *Aplysia depilans*, *Nucella lapillus*, *Patella depressa*, *Patella gomesii*, *Patella aspera*, *Patella* sp., *Umbraculum umbraculum*, *Patella ordinaria*, *Cerithium vulgatum*, *Onchidella celtica*), and fish (*Sphoeroides marmoratus*). The samples of *P. ordinaria* and *P. aspera* were purchased in local markets in Madeira, caught off the northern coast of the island (32°51′17.02″ N; 17°01′54.02″ W ). The organisms were transported to the laboratory in refrigerated containers. The samples that were not immediately processed were carefully stored at −20 °C.

### 4.2. Reagents

Pure TTX was purchased from Laboratorio CIFGA S.A. (Lugo, Spain). Ampoules contained 0.5 mL of solution with 25.9 ± 1.3 µg TTX/g and 2.99 ± 0.16 µg 4,9-anhydroTTX/g.

Acetonitrile, methanol and acetic acid were supplied by Panreac (Barcelona, Spain). All solvents employed in this work were HPLC or analytical grade and the water was distilled and passed through a water purification system (Milli-Q, Millipore, Mollet des Vallès, Spain). Formic acid was purchased from Merck (Darmstadt, Germany). Ammonium formate was from Fluka (Sigma-Aldrich, Madrid, Spain).

### 4.3. Sample Extraction

Samples were extracted on Silva et al. (2012) extraction protocol with appropriate amendments to the type of sample [16,23,45,46,47]. Animals were dissected and homogenized with a blender (A320R1, 700 W, Moulinex, Lisbon, Portugal) in pooled groups in order to obtain 1 g of tissue, with the exception of *M. glacialis*, *A. rubens*, *E. esculentus*, *C. lampas*, *O. ophidianus*, *U. umbraculum*, *P. lividus*, *S. granularis*, *D. africanum*, *A. depilans*, *H. sanctori* and *S. marmoratus* (here we divided the extraction into: gonads, muscle and liver). These species where treated individually, since each organism had enough extractable biomass. Samples were extracted in in 3 mL of acetic acid (1%)/methanol with the help of a vortex mixer for 5 min (Top Mix 1118, Fisher Bioblock Scientific) and ultrasonic bath, (5 min, 100 Hz) (RK100H, Bandelin SONOREX). A double extraction was performed, extracts were centrifuged at 4495× *g* for 15 min at 4°C (Centrifugal-Legend RT, Sorvall, Kendro Laboratory Products, Asheville, NC, USA), supernatants were combined and adjusted to a final volume of 7 mL. Then 1 mL of the extract was cleaned through a C18 solid-phase extraction (SPE), using cartridges (500 mg/3 mL volume from Supelco, Bellefonte, PA, USA) previously conditioned with 6 mL of methanol, followed by 6 mL of water (milliQ). The sample was eluted with 10 mL of 100% methanol and diluted with the same solvent to a final volume of 12 mL. Finally, each sample was concentrated by drying and re-suspending in 1 mL of methanol, and 500 µL were filtered through 0.22 µm filters (UltraFree-MC centrifugal devices, Millipore, Spain) before UHPLC-MS/MS analysis.

### 4.4. UHPLC Conditions

Chromatographic separation was carried out using a 1290 Infinity ultra-high-performance liquid chromatography system coupled to a 6460 Triple Quadrupole mass spectrometer (Agilent Technologies, Waldbronn, Germany). The toxins were separated using a column AQUITY UPLC BEH Amide (2.1 × 100 mm, 1.7 µm, Waters) at 35°C. Mobile phase A was 100% water with 10 mM formic acid and 10 mM ammonium formate. Mobile phase B was acetonitrile-water (95:5), containing 5 mM formic acid and 2 mM ammonium formate. The gradient program with a flow rate of 0.4 mL/min was started with 100% B and then a linear gradient to 65% B in 7 min. After an isocratic hold time linear of 2 min at 65% B and return to the starting conditions of 100% B in 0.5 min. Finally, 100% B was kept for 3.5 min before the next injection. The samples in the autosampler were cooled to 4°C and injection volume was 5 µL.

### 4.5. MS Methods

#### 4.5.1. Source Conditions

MS/MS detection was performed using an Agilent G6460C Triple Quadrupole mass spectrometer with an Agilent Jet Stream Electrospray source (Agilent Technologies, Waldbronn, Germany). Source conditions were optimized to achieve the best sensitivity for all compounds drying gas temperature of 250°C and drying gas flow 5 L/min, nebulizer gas pressure 55 psi (Nitrocraft NCLC/MS from Air Liquid), sheath gas temperature 400°C and sheath gas flow 12 L/min. The capillary voltage was set to 3000 V in positive mode with a nozzle voltage of 0 V. The fragmentor was 152 and the cell accelerator voltage was 2 for each TTX analogue in this method.

HRMS was acquired by IT-TOF mass spectrometer instrument from Shimadzu (Kyoto, Japan). The mass spectrometer was operated with the following conditions: a heat block and curve desolvation line temperature, 200°C; nebulizing gas flow, 1.5 L/min; detector voltage, 1.65 kV; and drying gas pressure, 105 kPa. The mass range was calibrated prior to data acquisition employing a direct infusion of IT-TOF standard sample.

The nitrogen generator was a Nitrocraft NCLC/MC from Air Liquid (Meicende, Spain).

#### 4.5.2. UHPLC-MS/MS Analysis: MRM Mode

The Agilent G6460C Triple Quadrupole mass spectrometer was operated in MRM in positive mode and the collision energy (CE) was optimized using MassHunter Optimizer software (Table 8). Two product ions were analyzed per compound, one for quantification and another for confirmation.

#### 4.5.3. UHPLC-MS/MS Analysis: PIS Mode

To confirm and identify TTX analogues, the fragmentation pathway for each molecule was used. The Agilent G6460C Triple Quadrupole mass spectrometer was operated in PIS in positive mode for each TTX analogue with a scan range from 50 to 350 *m*/*z*, scan time 120 msec and collision energy from 20 to 50 eV. The characteristic ions were always formed [M+H-H_2_O], *m*/*z* 162 and *m*/*z* 178/176 and sometimes also [M+H-2H_2_O].

#### 4.5.4. UHPLC-HRMS

HRMS was acquired using a UHPLC-MS-IT-TOF mass spectrometer instrument from Shimadzu (Kyoto, Japan) with a resolving power of 12,000 to identify TTX analogues. The mass spectra were acquired in full scan MS mode in positive mode with a mass range of 50–150 and 150–500 Da. The molecules were analysed using an ion accumulation time of 10 msec, with an event time of 100 msec and 1 repetition.

## Figures and Tables

**Table 1:** Tetrodotoxin and analogues molecular structure.

Structure	Analogue	R1	R2	R3	R4	Molecular Formula	Exact Mass
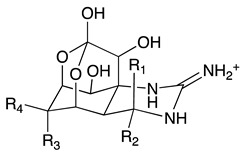	TTX	H	OH	OH	CH_2_OH	C_11_H_17_N_3_O_8_	320.1088
	4-*epi*TTX	OH	H	OH	CH_2_OH	C_11_H_17_N_3_O_8_	320.1088
	6-*epi*TTX	H	OH	CH_2_OH	OH	C_11_H_17_N_3_O_8_	320.1088
	11-deoxyTTX	H	OH	OH	CH_3_	C_11_H_17_N_3_O_7_	304.1139
	11-norTTX-6(R/S)-ol	H	OH	H	OH	C_10_H_15_N_3_O_7_	290.0983
	6,11-dideoxyTTX	H	OH	H	CH_3_	C_11_H_17_N_3_O_6_	288.1190
	11-oxoTTX	H	OH	OH	CHO	C_11_H_17_N_3_O_9_	336.1038
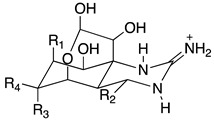	5-deoxyTTX	H	OH	OH	CH_2_OH	C_11_H_17_N_3_O_7_	304.1139
	5,11-dideoxyTTX	H	OH	H	CH_3_	C_11_H_17_N_3_O_6_	288.1190
	5,6,11-trideoxyTTX	H	OH	H	CH_3_	C_11_H_17_N_3_O_5_	272.1214
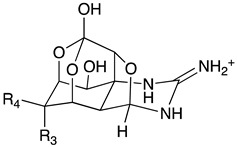	4,9-anhydroTTX	---	---	OH	CH_2_OH	C_11_H_15_N_3_O_7_	302.0983
	6-*epi*-4,9-anhydroTTX	---	---	CH_2_OH	OH	C_11_H_15_N_3_O_7_	302.0983
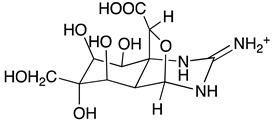	Tetrodonic acid	---	---	---	---	C_11_H_17_N_3_O_8_	320.1088

**Table 2:** TTXs analogues present in positive samples analyzed by MS/MS.

Suspicious TTXs	Multiple Reaction Monitoring (MRM)		Product Ions Scan (PIS)		
	Precursor Ion	Product Ions	[M+H]^+^	[M+H-H_2_O]^+^	Other Product Ions
oxoTTX	336	318/162	336	318	300/176/162
TTX and epimers	320	302/162	320	302	284/256/178/162
deoxyTTX	304	286/162	304	286	268/176/162
anhydroTTX	302	256/162	302	284	256/178/162
norTTX	290	272/162	290	272	176/162
dideoxyTTX	288	270/224	288	270	224/178/162
trideoxyTTX	272	254/162	272	254	236/178/162
anhydrotrideoxyTTX	254	236/162	254	236	178/162

**Table 3:** Sample information from continental Portugal. TTXs analogues in positive samples analyzed by high resolution mass spectrometry (HRMS) and quantified by MRM mode.

Sampling Date	Sampling Site	Sample	Species	Trophic Level [Ref]	Edible	Code	Suspicious TTXs	RT (min)	Elemental Composition	Theoretical *m*/*z*	Experimental *m*/*z*	Error (mDa)	TTX eq (µg/Kg)
September 2011	Porto Côvo	Limpet	*Patella depressa*	Grazer [27]	Yes	225	11-oxoTTX	7.2	C_11_H_17_N_3_O_9_	336.1038	336.1000	−3.8	<LOQ
		Gastropod	*Phorcus lineatus*	Grazer [28]	Yes	226	trideoxyTTX	7.7	C_11_H_17_N_3_O_5_	272.1241	272.1232	−0.9	<LOQ
October 2011	Pedra de Jacó	Sea-urchin	*Echinus esculentus*	Grazer [29]	Yes	229_2	11-oxoTTX	7.2	C_11_H_17_N_3_O_9_	336.1038	336.1020	−1.8	<LOQ
	Ovar	Gastropod	*Charonia lampas*	3rd Level Predator [30]	Yes	232_2	trideoxyTTX	7.7	C_11_H_17_N_3_O_5_	272.1241	272.1230	−1.1	<LOQ
						232_3	trideoxyTTX	7.7	C_11_H_17_N_3_O_5_	272.1241	272.1251	1.0	<LOQ
November 2011	Memória	Gastropod	*Nucella lapillus*	1st Level Predator [27]	Yes	236	trideoxyTTX	7.7	C_11_H_17_N_3_O_5_	272.1241	272.1239	−0.2	<LOQ
			*Phorcus lineatus*	Grazer [28]	Yes	239	11-oxoTTX	7.2	C_11_H_17_N_3_O_9_	336.1038	336.1000	−3.8	<LOQ
			*Gibbula umbilicalis*	Grazer [28]	Yes	240	trideoxyTTX	7.7	C_11_H_17_N_3_O_5_	272.1241	272.1238	−1.1	<LOQ

**Table 4:** Sample information São Miguel, Azores archipelago. TTXs analogues in positive samples analyzed by HRMS and quantified by MRM mode.

Sampling Date	Sampling Site	Sample	Species	Trophic Level [Ref]	Edible	Code	Standard TTX	Suspicious TTXs	RT (min)	Elemental Composition	Theoretical *m*/*z*	Experimental *m*/*z*	Error (mDa)	TTX eq (µg/Kg)
June 2013	Lagoa	Starfish	*Ophidiaster ophidianus*	Detritivorous [32]	No	412		norTTX	3.1	C_10_H_15_N_3_O_7_	290.0983	290.2489	150.6	30,902
								norTTX	4.6	C_10_H_15_N_3_O_7_	290.0983	290.2303	132.0	699
June 2013	Ilhéu de São Roque	Starfish	*Ophidiaster ophidianus*	Detritivorous [32]	No	424		norTTX	3.1	C_10_H_15_N_3_O_7_	290.0983	290.2475	149.2	42,604
								norTTX	4.6	C_10_H_15_N_3_O_7_	290.0983	290.2298	131.5	1638
June 2013	Caloura	Starfish	*Ophidiaster ophidianus*	Detritivorous [32]	No	435		norTTX	3.1	C_10_H_15_N_3_O_7_	290.0983	290.2478	149.9	20,853
June 2013	Caloura	Fish (Gonads)	*Sphoeroides marmoratus*	3rd Level Predator [33]	Yes	438 G	TTX		8.1	C_11_H_17_N_3_O_8_	320.1088	320.1088	0.0	352,886
							4-*epi*TTX		7.8	C_11_H_17_N_3_O_8_	320.1088	320.1082	−0.6	3157
								deoxyTTX	7.7	C_11_H_17_N_3_O_7_	304.1139	304.1129	−1.0	446
							11-deoxyTTX		7.0	C_11_H_17_N_3_O_7_	304.1139	304.1126	−1.3	157,156
								deoxyTTX	6.5	C_11_H_17_N_3_O_7_	304.1139	304.1124	−1.5	108,375
							4,9-anhydroTTX		7.1	C_11_H_15_N_3_O_7_	302.0983	302.1005	2.2	91
								dideoxyTTX	6.4	C_11_H_17_N_3_O_6_	288.1190	288.1157	−3.3	22,232
								trideoxyTTX	5.0	C_11_H_17_N_3_O_5_	272.1241	272.1198	−4.3	12,708
								trideoxyTTX	4.8	C_11_H_17_N_3_O_5_	272.1241	272.1228	−1.3	37,367
								trideoxyTTX	4.6	C_11_H_17_N_3_O_5_	272.1241	272.1227	−1.4	1849
								anhydrotrideoxyTTX	4.7	C_11_H_15_N_3_O_4_	254.1135	254.1122	−1.3	364
June 2013	Caloura	Fish (Liver)	*Sphoeroides marmoratus*	3rd Level Predator [33]	Yes	438 F	TTX		8.1	C_11_H_17_N_3_O_8_	320.1088	320.1098	1.0	41
							4-*epi*TTX		7.8	C_11_H_17_N_3_O_8_	320.1088	nd		6
							11-deoxyTTX		7.0	C_11_H_17_N_3_O_7_	304.1139	nd		<LOQ
								deoxyTTX	6.9	C_11_H_17_N_3_O_7_	304.1139	nd		4
								deoxyTTX	6.5	C_11_H_17_N_3_O_7_	304.1139	304.1153	1.4	136
							4,9-anhydroTTX		7.1	C_11_H_15_N_3_O_7_	302.0983	nd		<LOQ
								dideoxyTTX	6.4	C_11_H_17_N_3_O_6_	288.1190	nd		283
								trideoxyTTX	5.0	C_11_H_17_N_3_O_5_	272.1241	nd		31
								trideoxyTTX	4.8	C_11_H_17_N_3_O_5_	272.1241	272.1263	2.2	250
								trideoxyTTX	4.6	C_11_H_17_N_3_O_5_	272.1241	nd		14
June 2013	Caloura	Fish (Muscle)	*Sphoeroides marmoratus*	3rd Level Predator [33]	Yes	438 M	TTX		8.1	C_11_H_17_N_3_O_8_	320.1088	320.1088	0.0	9918
							4-*epi*TTX		7.8	C_11_H_17_N_3_O_8_	320.1088	320.1067	−2.1	118
								deoxyTTX	7.7	C_11_H_17_N_3_O_7_	304.1139	304.1164	2.5	29
							11-deoxyTTX		7.0	C_11_H_17_N_3_O_7_	304.1139	304.1172	3.3	48
								deoxyTTX	6.9	C_11_H_17_N_3_O_7_	304.1139	304.1123	−1.6	65
								deoxyTTX	6.5	C_11_H_17_N_3_O_7_	304.1139	304.1100	−3.9	2085
							4,9-anhydroTTX		7.1	C_11_H_15_N_3_O_7_	302.0983	302.0958	−2.5	45
								dideoxyTTX	6.4	C_11_H_17_N_3_O_6_	288.1190	288.1145	−4.5	2953
								trideoxyTTX	5.0	C_11_H_17_N_3_O_5_	272.1241	nd		122
								trideoxyTTX	4.8	C_11_H_17_N_3_O_5_	272.1241	272.1217	−2.4	507
								trideoxyTTX	4.6	C_11_H_17_N_3_O_5_	272.1241	nd		26
								anhydrotrideoxyTTX	4.7	C_11_H_15_N_3_O_4_	254.1135	254.1129	−0.6	5

nd—not detected.

**Table 5:** Sample information from the Moroccan coast. TTXs analogues in positive samples analyzed by HRMS and quantified by MRM mode.

Sampling Date	Sampling Site	Sample	Species	Trophic Level [Ref]	Edible	Code	Standard TTX	Suspicious TTXs	RT (min)	Elemental Composition	Theoretical *m*/*z*	Experimental *m*/*z*	Error (mDa)	TTX eq (µg/Kg)
July 2013	Mrizika	Gastropod	*Onchidella celtica*	Grazer [43]	No	474	TTX		8.1	C_11_H_17_N_3_O_8_	320.1088	320.1082	−0.6	12
							4-*epi*TTX		7.8	C_11_H_17_N_3_O_8_	320.1088	320.1071	1.7	8
								TTX epimer	7.5	C_11_H_17_N_3_O_8_	320.1088	nd		4
July 2013	Oualidia	Gastropod	*Aplysia depilans*	Grazer [44]	No	476	TTX		8.1	C_11_H_17_N_3_O_8_	320.1088	nd		<LOQ
							4-*epi*TTX		7.8	C_11_H_17_N_3_O_8_	320.1088	nd		<LOQ

nd—no detected.

**Table 6:** Coefficients of the Poisson model with ‘sampling site’ as factor.

Sampling Sites	Coefficents ± SE	z	*p*
**São Miguel**	8.96 ± 0.001	5000.3	<0.001
**Madeira**	−19.26 ± 21.38	−0.9	0.37
**Morocco**	−10.67 ± 0.38	−28.2	<0.001
**Continental Portugal**	−19.26 ± 13.30	−1.4	0.15

**Table 7:** Sampling sites and respective geographical coordinates, surveyed during September 2011 till January 2012, September 2012 and June and July 2013.

Date	Location	Sampling Site	Geographic Coordinates
September 2011	Continental Portugal	Luz	37°04′37″ N; 8°44′51″ W
		Camilo	37°05′14″ N; 8°40′06″ W
		Porto Côvo	37°53′33″ N; 8°47′38″ W
		Pelo Negro	41°19′22″ N; 8°71′69″ W
October 2011	Continental Portugal	Pedra de Jacó	41°17′14″ N; 8°70′78″ W
		Ovar	40°52′58″ N; 8°40′31″ W
		Esposende	41°29′5″ N; 8°46′45″ W
November 2011	Continental Portugal	Memória	41°13′50″ N; 8°43′18″ W
December 2011	Continental Portugal	Esposende	41°29′5″ N; 8°46′45″ W
		Valadares	41°5′29″ N; 8°39′27″ W
January 2012	Continental Portugal	Póvoa do Varzim	41°22′41″ N; 8°46′7″ W
		Angeiras	41°15′50″ N; 8°43′37″ W
September 2012	Madeira Island	Reis Magos	32°39′16″ N; 16°49′05″ W
		Caniçal	32°44′20″ N; 16°44′17″ W
June 2013	São Miguel Island	Cruzeiro	37° 50′31″ N; 25° 41′33″ W
		Étar de Ponta Delgada	37°44′19″ N; 25°39′38″ W
		São Roque	37°45′15″ N; 25°38′31″ W
		Mosteiros	37°53′25″ N; 25°49′14″ W
		Lagoa	37°44′42″ N; 25°19′47″ W
		Caloura	37°42′49″ N; 25°29′54″ W
July 2013	Morocco Coast	Casablanca corniche	33°36′01″ N; 7°39′57″ W
		El Jadida Haras	33°14′42″ N; 8°28′37″ W
		El Jadida Sâada	33°14′42″ N; 8°32′26″ W
		Sidi Bouzid	33°13′57″ N; 8°33′20″ W
		Mrizika	32°57′21″ N; 8°46′53″ W
		Oualidia	32°43′55″ N; 9°02′57″ W

**Table 8:** Main characteristics of UHPLC-MS/MS method in positive mode for TTX and detection of its analogues. The optimized transitions for each toxin are indicated in the table together with CE, fragmentor voltages, cell accelerator voltage (CAV) and polarity. Fragmentor voltage was 160 and CAV was 2 for each toxin.

Compound	Precursor Ion	Product Ion	CE
11-oxo-TTX	336	318	24
		178	40
TTX 4-*epi*TTX TTX epimers Tetrodonic acid	320		
		302	24
		161.9	36
	
11-deoxyTTX deoxyTTX	304	286	24
		176	40
4,9-anhydro-TTX anhydroTTX	302	256	28
		161.9	40
11-norTTX-6(R/S)-ol	290	272	24
		162	40
dideoxyTTX	288	270	24
		224	40
hydroxytrideoxyTTX	288	270	24
		162	40
trideoxyTTX	272	254	24
		162	40
hydroxyanhydrotrideoxyTTX	270	252	24
		162	40
anhydrotrideoxyTTX	254	236	24
		162	40
